# HMGA1-Regulating microRNAs Let-7a and miR-26a are Downregulated in Human Seminomas

**DOI:** 10.3390/ijms21083014

**Published:** 2020-04-24

**Authors:** Marco De Martino, Francesco Esposito, Simona Pellecchia, Ricardo Cortez Cardoso Penha, Gerardo Botti, Alfredo Fusco, Paolo Chieffi

**Affiliations:** 1 Istituto di Endocrinologia ed Oncologia Sperimentale–CNR c/o Dipartimento di Medicina Molecolare e Biotecnologie Mediche, Università degli Studi di Napoli “Federico II”, 80131 Naples, Italy; marco.demartino2@unina.it (M.D.M.); francesco.esposito2@unina.it (F.E.); simona.pellecchia@gmail.com (S.P.); 2 Department of Psychology, University of Campania “L. Vanvitelli”, 81100 Caserta, Italy; 3 Programa de Carcinogênese Molecular, Instituto Nacional de Câncer-INCA, Rua Andre Cavalcanti 37, Rio de Janeiro 20231-050, RJ, Brazil; ricardocortezcardoso@gmail.com; 4 Scientific Directorate, Istituto Nazionale Tumori–IRCCS–Fondazione “G. Pascale”, 80131 Napoli, Italy; g.botti@istitutotumori.na.it

**Keywords:** HMGA1, microRNAs, TGCTs, seminomas

## Abstract

Background: Recent studies have underlined HMGA protein’s key role in the onset of testicular germ cell tumors, where HMGA1 is differently expressed with respect to the state of differentiation, suggesting its fine regulation as master regulator in testicular tumorigenesis. Several studies have highlighted that the *HMGA1* transcript is strictly regulated by a set of inhibitory microRNAs. Thus, the aim of this study is to test whether HMGA1 overexpression in human seminomas may be induced by the deregulation of miR-26a and Let-7a—two *HMGA1*-targeting microRNAs. Methods: *HMGA1* mRNA and Let-7a and miR-26a levels were measured in a seminoma dataset available in the Cancer Genome Atlas database and confirmed in a subset of seminomas by qRT-PCR and western blot. A TCam-2 seminoma cell line was then transfected with Let-7a and miR-26a and tested for proliferation and motility abilities. Results: an inverse correlation was found between the expression of miR-26a and Let-7a and *HMGA1* expression levels in seminomas samples, suggesting a critical role of these microRNAs in *HMGA1* levels regulation. Accordingly, functional studies showed that miR-26a and Let-7a inhibited the proliferation, migration and invasion capabilities of the human seminoma derived cell line TCam-2. Conclusions: these data strongly support that the upregulation of HMGA1 levels occurring in seminoma is—at least in part—due to the downregulation of *HMGA1*-targeting microRNAs.

## 1. Introduction

Testicular germ cell tumors (TGCTs) afflict a wide age range of patients from children to young adults, and represent the most frequent cause of death due to cancer in this lifetime. TGCTs have their origin in a blocked maturation of a primordial germ cells (PGCs) [1], and more and more evidence reinforce the idea that the alteration of the epigenetic status is able to initiate human malignant germ cell tumors instead of somatic mutations. This makes clear the role of both genetic susceptibility and environmental factors in the TGCTs, named as “genvironment” [1]. TGCTs are classified into seminoma and non-seminoma germ cell tumors (NSGCTs), the last consisting in embryonal cell carcinoma, choriocarcinoma, yolk sac tumor (YST) and teratoma. Seminomas represent about 50% of all TGCTs, diagnosed in patients with a median age of 35 years, whereas NSGCTs arise prematurely at a median age of 25 years [2]. These latter can show several histological tumor elements, that is, the stem cell component embryonal carcinoma, teratoma (somatic differentiation), choriocarcinoma (extra-embryonic differentiation) and YST. The non-seminomas encompass about 40% of cases. Seminoma and non-seminoma components are the residual group and takes place at an intermediate age. Importantly, seminomas and NSGCTs show differentiated clinical features and deep divergences in therapy and prognosis [3,4].

The mammalian high mobility group A (HMGA) chromosomal protein family encompasses HMGA1a and HMGA1b, encoded by *HMGA1* gene via alternative splicing [5] and HMGA2 [6]. They are characterized by the ability to bind to DNA at AT-rich domains through their ‘AT-hooks’ regions. Even though HMGA members are chromatin-associated proteins, they do not have transcriptional activity *per se* but, by modifying the architecture of chromatin and participating in the assembly of multiprotein complexes with transcriptional factors, they can regulate gene transcription [7]. During embryogenesis *HMGA1* and *HMGA2* genes are highly expressed [8], whereas their expression is low or undetectable in normal adult tissues. However, several studies have demonstrated that their overexpression has an active role in malignant cell transformation. Indeed, thyroid cell transformation is prevented, and malignant cells are induced to death when HMGA expression is silenced [9,10]. Moreover, *in vitro* and *in vivo* observations have shown that HMGA proteins overexpression has an oncogenic activity, since effectively both HMGA1 and HMGA2 overexpression transforms mouse and rat fibroblasts [11], and both *Hmga1* and *Hmga2* transgenic mice develop NK-T cell lymphomas and pituitary adenomas [12,13,14]. We previously determined that mitotic cells (spermatogonia and primary spermatocytes) express HMGA1, instead in meiotic and postmeiotic cells (secondary spermatocytes and spermatids) HMGA2 is highly expressed [15,16]; in addition, we showed a specific role for HMGA2 in the spermatogenesis control. Indeed, we found that the spermatogenesis differentiation program is drastically compromised in *Hmga2*^−/−^ mice, regardless of the presence of *Hmga1* [16]. Lately, we demonstrated that the expression of HMGA has a key role in TGCT tumorigenesis and they can be considered a helpful diagnostic tool when the histological differential diagnosis is controversial [4,17]. Indeed, we demonstrated that HMGA expression is dependent on the state of differentiation of TGCTs: HMGA1 is overexpressed in seminomas, HMGA1 and HMGA2 are overexpressed in pluripotential embryonal carcinoma cells, and just HMGA2 is upregulated in YST, finally, the expression of both proteins is lost in mature adult tissue of teratoma areas [4,18].

However, even though it has been extensively demonstrated that HMGA proteins have a key function in neoplastic cell transformation, the pathways modulating HMGA protein levels remain mostly unknown. 

Recently, it has been proved that microRNAs (miRNAs) are able to regulate HMGA protein levels [19,20]. MiRNAs are a group of small noncoding RNAs that bind to the 3′-untranslated region (UTR) of the targeted mRNAs, thus causing mRNA degradation or the inhibition of its translation, regulating gene expression in a temporal and tissue-specific manner [21,22,23]. Really, in benign tumors of mesenchymal origin, *HMGA2* is frequently overexpressed due to the loss of its 3′-UTR that leads to the lack of miRNAs inhibitory effect [19,24], thus sustaining HMGA2 protein overexpression that then may account for cell transformation.

Therefore, the aim of our research work was to investigate whether HMGA1 overexpression, occurring in human seminomas, may be dependent on the deregulation of *HMGA1*-targeting miRNAs. In this manner, the overexpression of the HMGA proteins in seminomas can be, at least in part, explained by this hypothesis.

Starting from a set of already validated *HMGA1*-targeting miRNAs, we focused on the role of miR-26a and Let-7a in human seminomas. Indeed, these miRNAs were found decreased in human seminomas compared with the normal testis. Finally, functional assays confirmed that miR-26a and Let-7a inhibit the proliferation and motility of a seminoma cell line by targeting *HMGA1*, therefore suggesting a critical role of their downregulation in testicular tumorigenesis.

## 2. Results

**Figure 1:**
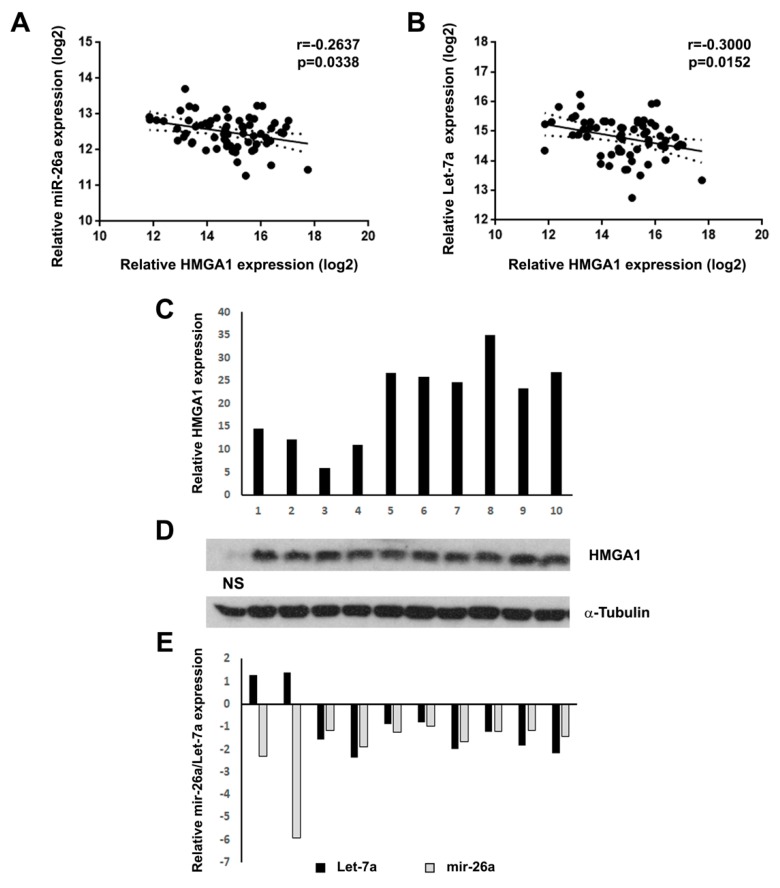
mir-26a and Let-7a are downregulated in seminoma and are negatively correlated with HMGA1. (**A**) Correlation scatter plot (Spearman’s Rank) analysis between mir-26a and *HMGA1* expression levels in the TCGA cohort (*n* = 65) (*r* = −0.2637; *p* = 0.0338). (**B**) Correlation scatter plot (Spearman’s Rank) analysis between Let-7a and *HMGA1* expression levels in the TCGA cohort (*n* = 65) (*r* = −0.3000; *p* = 0.0152). (**C**) qRT-PCR analysis of *HMGA1* mRNA expression in 10 seminoma samples. The fold change indicates the relative change in expression levels between seminoma and normal testis tissues, assuming that the mean value of three normal testis samples is equal to 1. (**D**) western blot analysis of HMGA1 protein expression in same testis samples analyzed in C. The level of α-tubulin was used as loading control. NS: Normal sample. (**E**) miR-26a and Let-7a qRT-PCR analysis in the same seminoma samples of C.

In the present work, we evaluated the correlation between *HMGA1* mRNA, Let-7a and miR-26a expression levels (Figure 1A,B) in a seminoma dataset available in the Cancer Genome Atlas (TCGA) database (*n* = 65) [25], since several studies [20,26,27,28,29] reported that *HMGA1* mRNA levels are negatively regulated by both Let-7a and miR-26a. Interestingly, both Let-7a and miR-26a levels were found negatively correlated with *HMGA1* expression levels, which our previous studies demonstrated to be upregulated in human seminoma [4], thus suggesting a negative control exerted by these miRNAs on *HMGA1* transcript in human seminoma (Figure 1A,B). To verify these data, HMGA1, Let-7a and miR-26a levels were assessed in a subset of seminomas and compared to normal samples by qRT-PCR and western blot analyses. Intriguingly, HMGA1 mRNA and protein levels were strongly upregulated in all the analyzed samples (Figure 1C,D), whereas Let-7a and mir-26a levels were decreased compared to normal samples (Figure 1E). These results suggest that the decrease in Let-7a and mir-26a levels may, at least partially, account for the HMGA1 enhanced levels in human seminoma.

**Figure 2:**
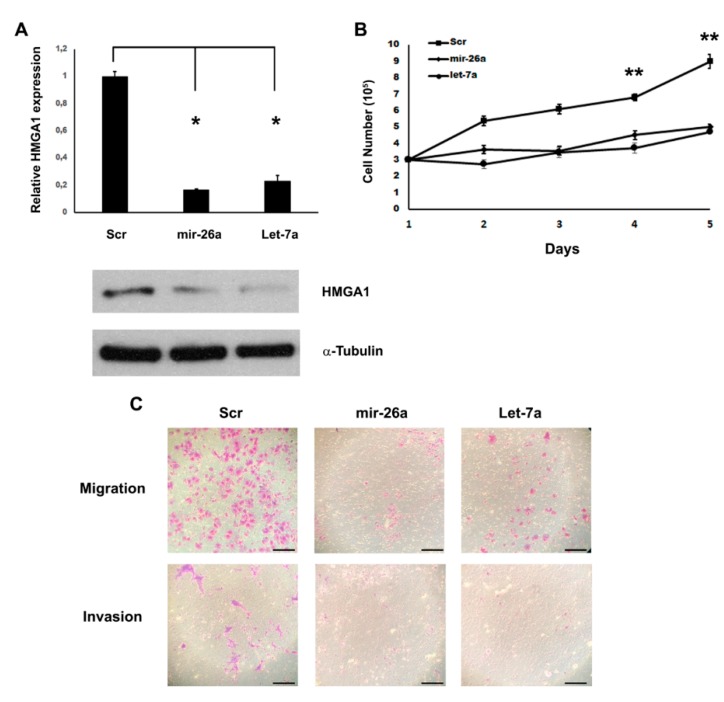
miR-26a and Let-7a inhibit cell proliferation and motility by targeting HMGA1. (**A**) qRT-PCR (upper panel) and immunoblot (lower panel) for HMGA1 after scrambled, mir-26a and Let-7a transfections. mRNA and proteins were extracted from the scrambled-, miR-26a-, Let-7a- transfected TCam-2 cells 48 h after transfection. For immunoblots the level of α-tubulin was used as loading control. The results are reported as the mean of expression values with error bars indicating SD; *n* = 4. * *p* < 0.05. (**B**) TCam-2 cells proliferation was assayed in miR-26a- and Let-7a-transfected cells, compared with scrambled-transfected cells. The cell proliferation results are reported as the mean of expression values with error bars indicating SD; *n* = 6. ** *p* < 0.01. (**C**) Migration and invasion capabilities were assayed in scrambled-, miR-26a- and Let-7a-transfected cells. Scale bar: 100 μm. A representative experiment is showed.

Indeed, it has been extensively demonstrated that these miRNAs are able to target *HMGA1* gene in osteosarcomas, lung adenocarcinomas, bladder cancers, breast cancers, pituitary tumors [20,26,27,28,29]. To test whether these miRNAs are able to target *HMGA1* also in seminoma, we transfected miR-26a and Let-7a into the seminoma cell line TCam-2 and we searched for fluctuations in HMGA1 amounts by western blot analysis. After the assessment of miR-26a and Let-7a overexpression in the TCam-2 transfected cells compared to the control, we found that the restoration of miR-26a and Let-7a resulted in the decrease of HMGA1 protein levels (Figure 2A). Moreover, a significant decrease in the *HMGA1* mRNA levels was also detected in the cells overexpressing these miRNAs (Figure 2A). These results validate the transcriptional regulation of the HMGA1 protein by miR-26a and Let-7a by promoting their role in *HMGA1* mRNA degradation.

To determine whether miR-26a and Let-7a downregulation affects seminoma cell growth, we restored their expression in the TCam-2 cell line by transient transfection. We found that, after the overexpression of miR-26a and Let-7a, TCam-2 cells grew at a notably slower rate in contrast with the scrambled vector-transfected cells (Figure 2B). Since HMGA1 is deeply involved in cell motility and miR-26a and Let-7a directly bind to its 3-UTR [20], we evaluated cell migration and invasion properties following the transient transfection of miR-26a and Let-7a by Transwell assays. Consistently with positive role of HMGA1 in cell motility, 24 h after plating, a substantial decrease of cell migration and, 48 h after plating, a noteworthy inhibition of cell invasion were detected in miR-26a- and Let-7a-overexpressing TCam-2 cells compared with controls (Figure 2C). These results indicate that the miR-26a- and Let-7a-mediated regulation of *HMGA1* can contribute to cell motility and invasion of the seminoma-derived cell line.

## 3. Discussion

Although different miRNA signatures are associated with histological subtypes of TGCTs, very few miRNAs have been found to have a key role in TGCTs. Voorhoeve et al. reported that two miRNAs (miR-372 and miR-373) can escape the cell cycle arrest exerted by p53 [30]. Indeed, these two miRNAs were not or scarcely expressed in TGCT-derived cell lines where p53 was mutated or downregulated, indicating that miR-372 and miR-373 induce TGCT growth eluding p53 checkpoint of cell-cycle. In this context, several data suggest that miR-372 and miR-373 may act as oncogenes in TGCTs through the inhibition of LATS2, a tumor suppressor gene [30].

Moreover, Ozata et al. demonstrated that *PEG3* mRNA can be strongly repressed by the action of miR-514a-3p, inducing apoptosis. In particular, PEG3 expression levels are increased in TGCTs, in which the expression of miR-514a-3p was lost [31].

Recent advances have reported that a deregulation of miRNA expression in cancer cells can modify tumor microenvironment, inducing cancer progression. However, this mechanism remains deeply unknown in TGCTs. Recently, it has been shown that epigenetic modifications downregulated miR-125b in TGCT samples. Indeed, xenograft models of TGCTs showed that miR-125b has a key role in tumor-stroma crosstalk, underlining its tumor-suppressor role and the possibility of using miR-125b as miRNA therapeutics [32].

During the last years, it has been progressively reported that miRNAs have key functions in gene modulation, cellular pathways, cancer features, such as the epithelial-to-mesenchymal transition and metastasis [33,34]. Indeed, miRNAs can work by regulating several target genes at the same time, acting as tumor-suppressor miRNAs through the downregulation of oncogenes or, on the other hands, as oncomirs by reducing the expression of tumor-suppressor genes [35].

Intriguingly, both Let-7a and miR-26a were found downregulated in several human cancer types, acting as tumor-suppressor miRNAs [36,37,38,39,40]. Moreover, these miRNAs inhibited cell proliferation and invasiveness of malignant melanoma derived-cell lines, suggesting that miR-26a and Let-7a may represent novel therapies for melanoma [41]. Indeed, Let-7 is able to repress several oncogenes such MYCN, AURKB, CCNF, RRM2, MKI67 and C12orf5 in TGCT [42].

Here, we show that Let-7a and miR-26a are downregulated in human seminoma negatively correlating with *HMGA1*. Then, we demonstrate that *HMGA1* is a target of Let-7a and miR-26a in seminomas and that they are able to inhibit seminoma cell growth and motility. Intriguingly, since miRNAs may act on several target transcripts that share the same microRNA Responsive Element (MRE) inhibitory action, it would be of great interest to study the transcriptomic effects exerted by Let-7a and miR-26a overexpression in seminoma-derived cell lines through RNA-seq analysis in order to obtain a broader investigation of overall gene expression changes (manuscript in preparation).

Currently, the study of the deregulated molecular pathways in TGCTs has led to the development of successful clinical approaches. Indeed, most of the TGCT patients are well treated by chemotherapy based on cisplatin. However, several patients developed chemoresistance to first line treatments for TGCTs. Therefore, new therapies based on novel strategies could increase the opportunity of treating cisplatin resistant patients and limit adverse drug reactions. Interestingly, the ability of Let-7a and miR-26a to prevent seminoma cell growth could open new therapeutic perspectives. Really, modern therapeutic methodologies may be founded on the reestablishment of the normal Let-7a and miR-26a levels in seminomas administrating synthetic miRNA oligonucleotides.

## 4. Materials and Methods

### 4.1. Cell Culture and Transfections

TCam-2 [43,44,45] cells were cultured in RPMI (Sigma-Aldrich, Saint Louis, MO, USA) supplemented with 10% fetal calf serum (Thermo Fisher, Waltham, MA, USA). To avoid mycoplasma contamination cells were regularly tested with MycoAlert (Lonza, Basel, Switzerland). TCam-2 cells were transfected using the Neon Transfection System MPK5000 (Thermo Fisher, Waltham, MA, USA) according to the manufacturer’s instructions. For transfection of miRNA oligonucleotides, cells were transfected with 50 nmol of miRNA precursors or control non-targeting scrambled oligonucleotides. Transfection efficiency was verified for each experiment by evaluating GFP expression.

### 4.2. Western Blotting

Protein extraction and western blotting procedures were carried out as reported elsewhere [46,47,48]. The antibodies used for western blotting were as follows: anti-α-Tubulin T518 (Sigma-Aldrich, Saint Louis, MO, USA). Anti-HMGA1 antibody was described elsewhere [49].

### 4.3. RNA extraction and qRT-PCR

Total RNA was extracted from tissues and cell cultures with Trizol (Life Technologies, Inc., Carlsbad, CA, USA) according to the manufacturer’s instructions. qRT-PCR analysis was performed by using miScript reverse transcription kit (QIAGEN, Hilden, Germany). cDNA was amplified by using miScript SYBR Green PCR kit (QIAGEN, Hilden, Germany), following manufacturer’s instructions. Reactions contained miScript Primer Sets (QIAGEN, Hilden, Germany), specific for each analyzed miR and U6 (used to normalize RNA levels). For *HMGA1* mRNA detection, we reverse transcribed total RNA from cells and tissues by using the QuantiTect reverse transcription kit (QIAGEN, Hilden, Germany), and then qRT-PCR for *HMGA1* was performed by using Power SYBR Green PCR Master Mix (Applied Biosystems, Foster City, CA, USA) and the following primers:

HMGA1-Fw 5′-aaggggcagacccaaaaa-3′ HMGA1-Rev 5′-tccagtcccagaaggaagc-3′

G6PD-Fw 5′-acagagtgagcccttcttcaa-3′ G6PD-Rev 5′-ataggagttgcgggcaaag-3′

### 4.4. Seminoma Tissue Samples

Neoplastic human seminoma tissues were obtained from surgical specimens and immediately frozen in liquid nitrogen. The samples were collected at the Biobanca Istituzionale dei Tessuti Istituto tumori Pascale, Naples, Italy as approved by Istituto Nazionale Tumori di Napoli, IRCCS “G. Pascale” in the Resolution of the Extraordinary Commissioner; number: 15, date: 20 January 2016. The tumor samples were frozen until required for RNA extraction. We declare that informed consent for the scientific use of biologic material was obtained from all patients.

### 4.5. Migration and Invasion Assay

Migration was assessed in Transwell chambers with a pore size of 5 µm (Corning, Corning, NY, USA). Cells (1 × 10^5^) were resuspended in serum-free medium before being plated in Transwell culture insert upper chamber. The bottom chamber was filled with culture medium supplemented with FBS 10%. After 24 hours of incubation, the cells remaining on the upper surface were wiped off with a cotton swab. The cells that migrated to the underside of the Transwell filters were fixed and stained with crystal violet (Sigma-Aldrich, Saint Louis, MO, USA). Invasion assays used identical methods, except that the cells were placed in the top compartment of a modified Boyden chamber on a Matrigel-coated membrane.

### 4.6. Statistical Analysis

Data were analyzed using a two-sided unpaired Mann–Whitney test (GraphPad Prism, GraphPad Software, Inc., San Diego, CA, USA). Values of *p* < 0.05 were considered statistically significant. The mean +/− s.d. of three or more independent experiments is reported. The correlation analyses were evaluated through non-parametric Spearman’s Rank correlation and generated using GraphPad Prism, GraphPad Software, Inc.

## 5. Conclusions

In conclusion, the present work defines *HMGA1* as a target of Let-7a and miR-26a in seminomas and then their down-regulation in these tumors can contribute to the increased HMGA1 protein levels described by our previous research, adding another piece to understanding the mechanisms that are involved in the genesis of human seminomas.
